# Progesterone Receptor Activates Msx2 Expression by Downregulating TNAP/Akp2 and Activating the Bmp Pathway in EpH4 Mouse Mammary Epithelial Cells

**DOI:** 10.1371/journal.pone.0034058

**Published:** 2012-03-22

**Authors:** Jodie M. Fleming, Erika Ginsburg, Anita S. Goldhar, Joshua Plant, Barbara K. Vonderhaar

**Affiliations:** 1 Mammary Biology and Tumorigenesis Laboratory, Center for Cancer Research, National Cancer Institute, Bethesda, Maryland, United States of America; 2 Department of Biology, North Carolina Central University, Durham, North Carolina, United States of America; Ecole Normale Supérieure de Lyon, France

## Abstract

Previously we demonstrated that EpH4 mouse mammary epithelial cells induced the homeobox transcription factor Msx2 either when transfected with the progesterone receptor (PR) or when treated with Bmp2/4. Msx2 upregulation was unaffected by Wnt inhibitors s-FRP or Dkk1, but was inhibited by the Bmp antagonist Noggin. We therefore hypothesized that PR signaling to Msx2 acts through the Bmp receptor pathway. Herein, we confirm that transcripts for Alk2/ActR1A, a non-canonical BmpR Type I, are upregulated in mammary epithelial cells overexpressing PR (EpH4-PR). Increased phosphorylation of Smads 1,5, 8, known substrates for Alk2 and other BmpR Type I proteins, was observed as was their translocation to the nucleus in EpH4-PR cells. Analysis also showed that Tissue Non-Specific Alkaline Phosphatase (TNAP/Akp2) was also found to be downregulated in EpH4-PR cells. When an Akp2 promoter-reporter construct containing a ½PRE site was transfected into EpH4-PR cells, its expression was downregulated. Moreover, siRNA mediated knockdown of Akp2 increased both Alk2 and Msx2 expression. Collectively these data suggest that PR inhibition of Akp2 results in increased Alk2 activity, increased phosphorylation of Smads 1,5,8, and ultimately upregulation of Msx2. These studies imply that re-activation of the Akp2 gene could be helpful in downregulating aberrant Msx2 expression in PR+ breast cancers.

## Introduction

Progesterone (P) is a key regulator of mammary gland proliferation and differentiation. The action of progesterone is mediated by two isoforms of the progesterone receptor (PR), which are temporally and spatially regulated during mammary development in association with their precise functions [1]. The A isoform (PR-A) is exclusively expressed in the virgin gland and has been associated with ductal elongation and secondary branching, while the B isoform (PR-B) is abundantly expressed during pregnancy and is associated with lobuloalveolar development [2]. Transgenic mice that overexpress PR-A demonstrate extensive lateral side-branching at 10–14 weeks of age compared to wild type controls [2]. Additionally, EpH4 cells are a normal mammary epithelial cell line derived from mammary gland of a mouse in the mid-gestation stage [3] and studies [4], [5] have shown that PR is downregulated at this stage. Previously, we showed that stable transfection of PR-A into EpH4 mouse mammary epithelial cells enhanced branching morphogenesis on collagen gels through upregulation of transcription of the homeobox gene Msx2 [6]. Consistent with these results, our transgenic mice overexpressing Msx2 in the mammary gland demonstrated extensive lateral branching postnatally compared to wild type controls [6]. Since there is no progesterone responsive element (PRE) on the Msx2 promoter [7], regulation must be indirect through other transcription factors. While others have shown that P-dependent side-branching is enhanced by Wnt 4 [8], we found no evidence that the Wnt pathway was involved in Msx2 expression or branching of the cells [6]. However, we did find that treating the parental EpH4 cells with Bone Morphogenic Protein 2 (Bmp2) or 4 (Bmp4) induced both Msx2 expression and branching morphogenesis, suggesting that a signaling cascade starting from PR and ending with Msx2 occurred through the Bmp pathway.

Bmps were originally identified by their ability to cause bone differentiation, but are now known to be major players in the regulation of embryonic development and postnatal homeostasis of various organs and tissues, by controlling cellular differentiation, proliferation and apoptosis [9]. In the embryo, Bmp4, Msx2, phospho-Smad 1, and Bmp receptor type 1A (BmpR1A) are co-localized in the ventral ectoderm and collectively regulate proper hind limb formation [10]. Msx2 has also been shown to co-localize with the Bmps in the mouse mammary gland [11]. While our previous study [6] showed that the Bmp pathway was involved in induction of Msx2 in mammary cells, we found no evidence either by RT-PCR or western blot analysis that either of the closely related Bmp2 or 4 [11] were upregulated in the EpH4-PR cells, which have increased Msx2 expression. Therefore, to investigate the interactions of Bmp and PR signaling with Msx2 expression, we examined expression of BmpR and downstream components of the signaling pathway in the mouse mammary EpH4-PR cells.

The BmpR 1 in EpH4 cells is Alk2/ActR-1A. Alk2, as other receptors of the TGFβ superfamily, is a transmembrane receptor with intrinsic cytoplasmic serine/threonine kinase activity [12]. It is found in the embryo [9], [13] and in the developing neonate lung [14]. Alk2, while not a canonical BmpR, binds both activin and Bmp2/4 in conjunction with corresponding type II receptors [13]. Upon ligand binding, type II receptors phosphorylate type I receptors in the GS domain. Type I receptors then bind and phosphorylate the Smad proteins [15]. Smad 1, 5, and/or 8 complex with Smad 4, and migrate to the nucleus where they act as transcription factors [12]. In association with this pathway, Tissue Non-Specific Alkaline Phosphatase (TNAP/Akp2) has been shown to negatively regulate Alk2 [16]. Herein, we report a significant downregulation of Akp2 expression in the EpH4-PR cells through PR regulation of its promoter. This downregulation of Akp2 results in upregulation of Alk2, phosphorylation of the Smads, and increased Msx2 expression, thereby linking PR to Msx2 induction.

## Methods

### Cell Culture and Reagents

EpH4 cells, a mouse mammary epithelial cell line [17], were transfected and clones selected as previously described [6]. EpH4-EV or EpH4-PR cells were routinely maintained in DMEM Growth Media (DMEM/GM) containing 10% FBS, 2 mM L-glutamine (Invitrogen, Gaithersburg, MD), 20 mM HEPES (Sigma, St. Louis, MO), penicillin (100 U/ml) and streptomycin (100 µg/ml) and neomycin (G418; 400 µg/ml). For hormone or growth factor treatment, cells were quiesced for 6 to 24 hours with a minimal medium containing 5% charcoal stripped serum (CSS; Gemini Bio-Products, West Sacramento, CA; DMEM/CSS). Prior to treatment, cells were plated in 6-well dishes at a density of 100,000 cells/ml for RNA extraction and 120,000 cells/ml for total protein extraction. For nuclear protein experiments, cells were plated at a density of 100,000 cells/ml in T150 flasks. Cells were grown to approximately 70% confluence in DMEM/GM, and then rinsed in PBS, and media changed to DMEM/CSS with either 10^−8^ M progesterone (P; Sigma), Noggin (10 nM, R&D Systems, Minneapolis, MN), Bmp2 or Bmp4 (R&D Systems, Minneapolis, MN) at a final concentration of 300 ng/ml.

### Microarrays

Microarrays were performed and analyzed as previously described [18] by Cogenics, Inc. (Morrisville, NC) on RNA collected from EpH4-EV or EpH4-PR cells grown in DMEM/GM. Data was subjected to functional analysis through the use of Ingenuity pathways analysis (IPA; Ingenuity Systems, http://www.ingenuity.com). Microarray data with its associated MIAME compliant data has been deposited in the public repository, Gene Expression Omnibus, accession number GSE30637.

### Semi-quantitative RT-PCR

Total RNA was extracted using TRIzol Reagent (Invitrogen), treated with DNase (Invitrogen) to remove potential genomic contamination, and reverse transcribed as previously described [6]. For all PCR analyses, the number of cycles used was selected at the linear expression level for each gene tested. PCR products were resolved by gel electrophoresis, quantified using NIH Image J and expressed relative to the corresponding housekeeping gene. PCR primers used and conditions for amplification were as outlined in Table 1A. Gene expression levels were calculated relative to the housekeeping gene actin or GAPDH for each experiment.

**Table 1:** PCR primer sequences and conditions.

A. RT-PCR Primers	Primer sequence (5′– 3′)	Anneal temp, # of cycles
Alk2 forward	GGAGAAGTATGGAGGGGCAGCT	59°, 30
Alk2 reverse	GGCCCAAATATCGACCCTCTTA	
Akp2 forward	AGTCCGTGGGCATTGTGACTA	60°, 30
Akp2 reverse	CCACCTATGATCACGTGAT	
BmpR1A forward	ACCATTTCCAGCCCTACATC	52°, 30
BmpR1A reverse	TTTCACACACACAACCTCAC	
Actin forward	GTGGGCCGCTCTAGGCACCAA	60°, 21
Actin reverse	CTCTTTGATGTCACGCACGATTTC	
GAPDH forward	CATGTGGGCCATGAGGTCCAC	60°, 20
GAPDH reverse	TGAAGGTCGGAGTCAACGGATTTGGT	

B. Cloning Primers	Primer sequence (5′– 3′)	Anneal temp, # of cycles
Akp2 promoter forward	CCGGAAGCTTAGACCGAGTTTGGAACATGC	60°, 35
Akp2 promoter reverse	CCGGCTCGAGGCTCCGTGGTAAGAGACAGG	

### Cloning of the Akp2 promoter

The Akp2 promoter was amplified from a BAC clone (RP23-400F8, Accession # AL807764N; Children's Hospital Oakland Research Institute, Oakland, CA) and PCR cloned into a pGL3 basic vector (Promega, Madison, WI) with PCR Extension kit (5 Prime, Gaithersburg, MD) at the XhoI and HindIII restriction sites. The Akp2 promoter construct contains a ½ PRE site at −1495 to −1501 bp [19]. Primers (Table 1B) were used at a 300 nM final concentration using 50 ng of the BAC clone. PCR products were resolved on a 1% agarose gel, excised, and extracted with a MinElute Gel Extraction kit (Qiagen, Valencia, CA). Purified products were digested with XhoI/HindIII and ligated into pGL3 with T4 Rapid Ligation kit (Roche Applied Science, Indianapolis, IN), purified, cloned, and sequence verified.

### siRNA mediated knockdown of AKP2

Non-targeting and AKP2 siRNA pools were purchased from Dharmacon (Lafayette, CO). EpH4-EV cells were transfected with 100 nM siRNA using DharmaFECT1 following manufacturers instructions. Cells were harvested 24 and 48 hours post-transfection.

### Luciferase Reporter Assay

EpH4-EV or EpH4-PR cells were plated in 35 mm dishes at a density of 200,000 cells/ml in DMEM/GM. After overnight attachment, each well was transiently transfected using FuGENE6 (Roche Applied Science) with 100 ng pRL-TK construct to normalize for transfection efficiency and pGL3 basic vector containing 1 µg Akp2 promoter construct. The following day, media were replaced with DMEM/CSS in the presence or absence of 10^−8^ M P. After an additional 24 hour, cells from replicate wells were collected in 1× Reporter Lysis Buffer (Luciferase Assay System, Promega) and assayed for luciferase activity on a Lumat LB9507 luminometer.

### Western Blot Analysis

EpH4-EV or EpH4-PR was collected, homogenized (50 mM Tris-HCl, pH 7.5, 50 mM sodium chloride, 1 mM DTT, 1 mM PMSF, 10 µg/ml leupeptin, 10 µg/ml aprotinin, 1 µg/ml pepstatin and 0.1 mM sodium orthovanadate), sonicated, and protein concentration determined [20]. Proteins were subjected to 10% SDS-PAGE, transferred to Hybond nitrocellulose ECL membrane and probed with antibodies for Alk2, Smad 8 (Santa Cruz Biotechnologies, Santa Cruz, CA); phospho-Smad 1/5/8, phospho-Smad 1/5, Smad 5 or Smad 4 (Cell Signaling, Beverly, MA), or BMPR1A (AbCam, Cambridge, MA). Tubulin was used as loading control (Sigma). All antibodies were used at concentrations recommended by the manufacturers. Immunoreactivity was determined using enhanced chemiluminescence (ECL Plus; GE Healthcare, Pittsburgh, PA).

### Preparation of Nuclear Extracts

Cells were plated as outlined above for protein isolation. Cells were collected, resuspended, and lysed as previously described [21]. Nuclear extracts were frozen at −80°C until ready for use.

### Cross-linking to Protein A beads

Protein A-agarose beads (Santa Cruz Biotechnology) were washed and incubated with Smad 4 antibody for 1.5 hours at room temperature. Beads were washed twice with 0.2 M sodium borate, pH 9, then mixed, end-over-end, with 5.2 mg/ml dimethyl pimelimidate (Sigma) in 0.2 M sodium borate, pH 9, for 1 hour at room temperature, washed with 0.2 M ethanolamine (Sigma), and finally resuspended in PBS.

Nuclear extracts (1 mg) were added to the cross-linked beads and incubated for 1.5 hours at 4°C while mixing. Beads were washed in buffer containing protease inhibitors, collected, resuspended in Tris-Glycine SDS Sample Buffer (Invitrogen) and boiled. Resulting supernatant was loaded onto a gel for western blot analysis, probed with phospho Smad 1,5,8 antibody, then stripped and reprobed with Smad 5 antibody.

### Fluorescent immunocytochemistry

EpH4-EV and EpH4-PR cells were plated on 8-well glass chamber slides (Nunc, Rochester, NY) at either 10,000 or 20,000 cells/chamber in DMEM/GM. The following day, media were changed to DMEM/CSS in the presence or absence of 10^−8^ M P. Three hours later, cells were fixed in ice cold 100% methanol, washed in PBS containing 0.1% Triton X-100, and blocked in 5% normal goat serum (Jackson Laboratories, Bar Harbor, ME) overnight at 4°C. Slides were then incubated with 1 µg/ml phospho-Smad 1,5, 8 for 1 hour at room temperature. In all cases no primary antibody served as the negative control. Slides were washed four times with PBS-0.1% Triton followed by incubation for 1 hour with red fluorescent tagged goat anti-rabbit secondary antibody (AlexaFluor 594, 1∶500, Invitrogen) in the dark. After extensive washing with PBS containing Triton, slides were mounted with Prolong Gold antifade reagent with DAPI (Invitrogen). The fluorescent staining pattern was evaluated using an Olympus BX40 fluorescence microscope (Olympus America, Center Valley, PA).

### Statistics

Images were quantified using NIH Image J64 software. The statistical significance of the difference between groups was determined via Student's *t*-test using GraphPad InStat Software version 3.0 b (San Diego, CA). Data are expressed as the mean ± SD. P≤0.05 was considered significant.

## Results

### Progesterone receptor signaling modulates the Bmp pathway in EpH4 cells

In our previous study [6] we showed that EpH4 cells expressing PR-A (EpH4-PR) upregulate expression of the homeobox-containing transcription factor Msx2, and demonstrate enhanced branching on collagen gels. While our data also suggested that the Bmp signaling pathway was involved in the process, we were unable to demonstrate that expression of the ligands, Bmp2 and Bmp4, was altered in the EpH4-PR cells compared to the control cells transfected with the vector alone (EpH4-EV). To further explore the differences in these cells, microarray analysis was performed using Agilent whole mouse genome arrays (*data not shown*). Pathway analysis suggested that a constitutively active BmpR 1, resulting from a decrease in expression of the phosphatase TNAP/Akp2, might be responsible for the Msx2 upregulation.

**Figure 1:**
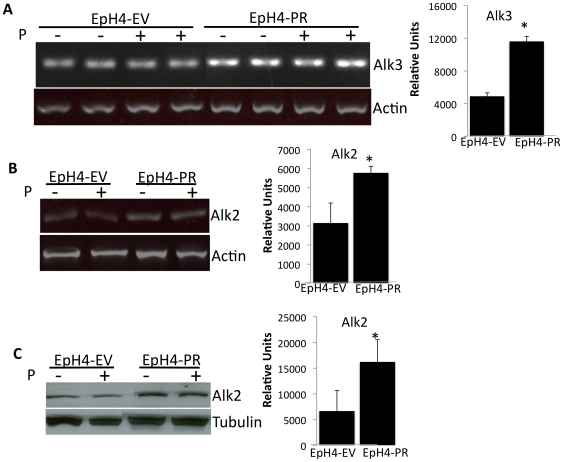
Progesterone Receptor stably transfected into mammary epithelial cells upregulates expression of canonical BmpR 1A (Alk3) and non-canonical BmpR Activin-Like Kinase Receptor ActR1A (Alk2). EpH4 cells stably transfected with empty vector (EV) or the progesterone receptor (PR) were treated+/−10^−8^ M P for 24 hours. Total RNA was isolated and transcript abundance was evaluated via PCR for either Alk3 (A) or Alk 2 (B). Actin was used as housekeeping gene. C) EpH4-EV and EpH4-PR cells were plated at 2.4×10^5^ cells/well in 6 well dishes and treated the next day+/−10^−8^ M P for 24 hours. Total cell lysates were analyzed via western blotting with the indicated antibodies then stripped and re-probed for tubulin as a loading control. Representative blots of at least three experiments are shown. Graphs represent mean+/−SD of at least three independent experiments, * P<0.05.

The canonical Type 1A BmpRs are Activin-like kinases 3 and 6 (Alk3 and Alk6). By semiquantitative RT-PCR, we found that EpH4-PR cells, which constitutively express PR-A and Msx2, upregulate BmpR1A/Alk3 mRNA relative to control EpH4-EV cells (Fig. 1A). However, we were unable to detect BmpR1A protein in the EpH4-PR cells by western blot analysis (*data not shown*), though mammary tissue was successfully used as a positive control for Alk3 detection. Interestingly, the non-canonical Bmp receptor ActR-1A/Alk2 is also upregulated in the EpH4-PR cells as determined by RT-PCR (Fig. 1B). Western blot analysis confirmed that Alk2 protein expression is greater in EpH4-PR cells than in the EpH4-EV cells (Fig. 1C), suggesting that this receptor may be the main effector of PR signaling through the Bmp receptor pathway in our EpH4-PR cells.

### EpH4-PR cells or EpH4 parental cells treated with Bmp2 or Bmp4 upregulate Smad proteins and phosphorylation

**Figure 2:**
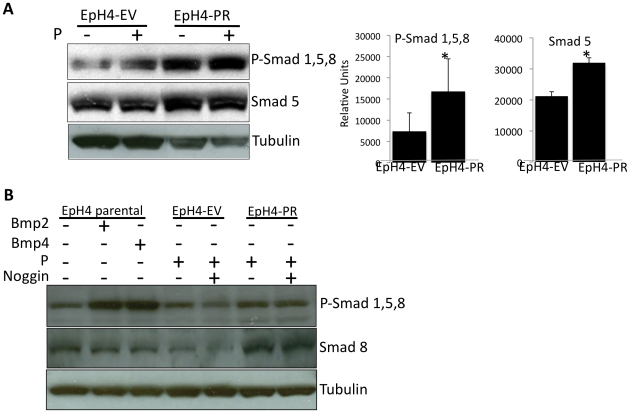
Phospho-Smads are upregulated in progesterone receptor expressing mammary epithelial cells. A) EpH4 cells stably transfected with empty vector (EV) or the progesterone receptor (PR) were treated+/−10^−8^ M P for 6 hours. Total cell lysates were analyzed via western blotting with phospho-Smad 1,5 and then stripped and re-probed for Smad 5 or tubulin. Graphs represent mean+/−SD of at least three independent experiments, * P<0.05. B) EpH4 parental cells were treated+/−300 ng/ml Bmp2 or Bmp4 for 6 hours. EpH4-EV and -PR cells were treated+/−10^−8^ M P in the presence or absence of 10 nM Noggin for 6 hours. Total cell lysates were analyzed via western blotting with phospho-Smad 1,5, 8, Smad 8 or tubulin antibodies. The blot was stripped between assays.

To confirm that that BMP-receptor signaling is activated by PR-signaling in EpH4 cells, we examined the levels and activation of the Smad proteins, downstream transcription factors of the Bmps. As shown in Fig. 2A, the levels of both phospho-Smad 1,5 and Smad 5 are significantly increased in the EpH4-PR cells relative to EpH4-EV cells (*P<0.05*), even in the absence of exogenous P. This increase is believed to be due to the ability of PR-A to act in an unliganded manner, as observed in other studies [22]–[24]. No significant increase in Smad phosphorylation was observed upon treatment with 10^−8^ M P in either cell line. Consistent with previous results [6], the phosphorylation of Smad 1,5, 8 is increased in EpH4 parental cells that have been treated with Bmp2 or Bmp4 (Fig. 2B). Smad 8 was also upregulated in EpH4-PR cells relative to EpH4-EV cells. While we have seen Noggin suppress Msx2 in the EpH4-PR cells [6], addition of Noggin (10 nM) minimally decreases phosphorylation of Smad 1,5, 8 in EpH4-EV cells, but not in PR cells. This may suggest that Bmps binding to Bmp receptors is not required for activation of BMP-receptor signaling in response to PR signaling.

### Smads 1, 5, and 8 translocate preferentially to the nucleus in EpH4-PR cells

**Figure 3:**
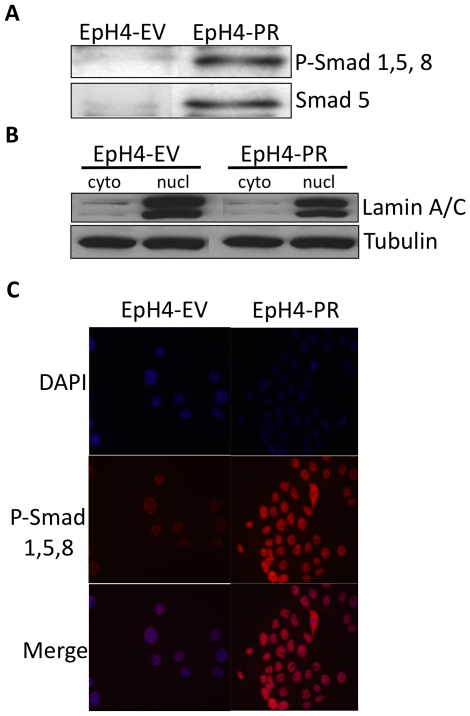
Smad 1, 5, 8 translocate preferentially to the nucleus in mammary epithelial cells expressing the progesterone receptor. EpH4 cells stably transfected with empty vector (EV) or the progesterone receptor (PR) were quiesced by overnight incubation in media containing charcoal stripped serum. A) Nuclear extracts were isolated and immunoprecipitated with Smad 4 antibody that had been cross-linked to Sepharose A beads, separated on a 10% Tris-Glycine gel, and probed with either phospho (P) -Smad 1,5, 8 or Smad 5 antibodies. B) Non-immunoprecipitated samples of cytosolic and nuclear fractions from EpH4-EV and -PR cells were separated and probed with Lamin A/C or tubulin to assure purity of nuclear preparations and equal loading, respectively. cyto = cytosolic, nucl = nuclear. C) EpH4 cells stably transfected with empty vector (EV) or the progesterone receptor (PR) were treated with 10^−8^ M P for 24 hours, fixed and then subjected to fluorescent immunocytochemistry. Images are representative immunofluorescent staining of phospho-Smad 1, 5, 8 (red) and nuclei were stained with DAPI (blue).

When Smads 1, 5, and/or 8 are phosphorylated, they bind with the common Smad 4 and translocate to the nucleus for initiation of transcription [12]. To evaluate differences in amount of activated nuclear Smads between the EpH4-EV and EpH4-PR cells, nuclear extracts were isolated and the levels of Smads were determined via immunoprecipitation and western analysis. Immunoprecipitated nuclear extracts of EpH4-EV and EpH4-PR cells with Smad 4 antibody demonstrated higher level of phospho Smad 1,5, 8 and Smad 5 in the EpH4-PR cells (Fig. 3A). Treatment with 10^−8^ M P had no significant effect above results observed with untreated cells. The quality of the nuclear and cytoplasmic preparations is shown in Fig. 3B. Enhanced nuclear localization of Smads in EpH4-PR cells was further confirmed by fluorescent immunocytochemistry (Fig. 3C).

### Tissue Non-Specific Alkaline Phosphatase (Akp2) is downregulated in EpH4-PR cells

**Figure 4:**
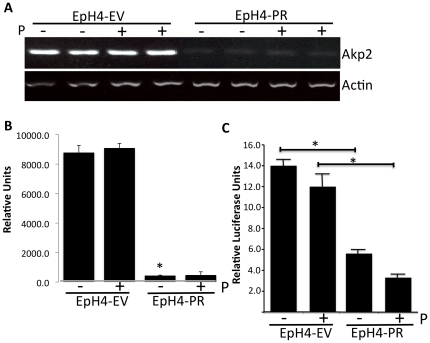
TNAP/Akp2 is downregulated in progesterone receptor overexpressing mammary epithelial cells. A) RNA was isolated from EpH4 cells stably transfected with empty vector (EV) or the progesterone receptor (PR) and transcript abundance was analyzed via semi-quantitative PCR. B) Quantitation of transcript abundance. Data represent the mean+/−SD, * P<0.05. C) Luciferase Assay for Akp2 promoter. EpH4-EV and EpH4-PR cells were transfected with an Akp2 promoter-luciferase construct and with the internal control vector, pRL-TK and 24 hours after transfection, media were removed and treated with or without 10^−8^ M P. Twenty-four hours later, triplicate samples were collected and analyzed. Relative luciferase units are reported as means+/−SE. * *P<0.05* between EpH4-EV and EpH4-PR cells.

Alk2 can be dephosphorylated by the phosphatase Akp2 [16] and our microarray analyses showed downregulation of Akp2 in EpH4-PR cells (*data not shown*). Thus, the downregulation of Akp2 may explain the upregulation of Alk2 and Smad phosphorylation even in the presence of Noggin. To confirm this result, the transcript abundance of Apk2 was measured in the EpH4-EV and -PR cells and, correspondingly, Akp2 was significantly downregulated in EpH4-PR cells (*P<0.05*, Fig. 4A/B). Treatment with P did not significantly alter Akp2 levels from the effects observed by unliganded PR. Downregulation of this phosphatase suggests that its substrates such as BmpR I or, in our case, Alk2, are constitutively activated. Bmps, therefore, would not be required for observed upregulation and phosphorylation of the Smads in the EpH4-PR cells. We investigated whether PR acts either directly or indirectly on the Akp2 promoter to suppress its activity. A portion of the PR that includes the ½ progesterone response element (PRE) site (−1495 to −1501; [19], [25]) was cloned and the construct transfected into EpH4-EV and EpH4-PR cells. Results demonstrate a significant downregulation of expression from the Akp2 promoter in EpH4-PR cells (Fig. 4C, *P<0.05*). Treatment with 10^−8^ M P further represses expression driven from the promoter in EpH4-PR cells to 23% of the level found in similarly treated control EpH4-EV cells. These data show that constitutive activation of the Alk2 receptor may be the result of PR downregulation of Akp2 promoter through the ½ PRE site.

### Depletion of Tissue Non-Specific Alkaline Phosphatase (Akp2) results in upregulation of Alk2 and Msx2

**Figure 5:**
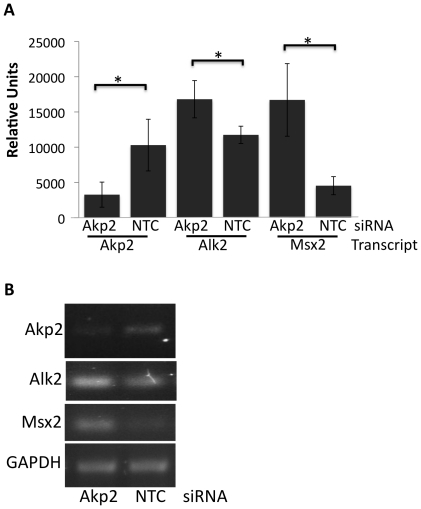
Depletion of endogenous TNAP/Akp2 results in upregulation of Alk2 and Msx2 expression. EpH4 cells were transfected with non-targeting or AKP2 siRNA. Twenty-four to 48 hours post-transfection, total RNA was isolated and transcript abundance was analyzed via semi-quantitative PCR. A) Data represent the mean+/−SD of four independent experiments. B) Representative images from PCR products. NTC = non-targeting control siRNA. *P<0.05.

Our current and previous results collectively suggest that expression of PR in mammary epithelial cells downregulates Akp2 and that suppression of Akp2 results in an increase in Alk2 and Msx2 expression and invasive cell behavior [6]. Therefore, siRNA-mediated knockdown of Akp2 was used to directly test the ability of Akp2 to regulate Alk2 and Msx2 expression. Twenty-four hours post-transient transfection using a pool of siRNA targeting Akp2 yielded a 70% increase in Akp2 expression, and 48-hours post-transfection a significant increase in both Alk2 and Msx2 expression was observed (Fig. 5, *P<0.05*). These data demonstrate the direct ability of Akp2 to regulate Alk2 and Msx2.

## Discussion

The conflicting roles of P in breast cancer etiology highlight the importance of understanding the consequences of PR signaling in mammary epithelial cells. The data presented herein, along with other clinical *in vitro* and *in vivo* analyses suggests that activation of P signaling leads to increased cell proliferation and events leading to aggressive cell behaviors [6], [26]–[30]. In the normal breast, PR-A and -B are co-expressed in the same cells; however, in breast cancer the PR-A: PR-B ratio is higher and PR-A is associated with a less differentiated and more aggressive tumor [1], [22]. Our data and previous studies show that PR-A can act in an unliganded fashion to activate gene transcription in breast cancer cells [22]–[24], and unliganded PR was recently shown to attenuate taxane-induced apoptosis [23]. PR-A expression in EpH4 cells did not increase cell growth on plastic, but did increase branching morphogenesis via Msx2 induction [6], [21]. Our results demonstrating PR-BMP-Smad signaling leading to Msx2 expression and increased branching morphogenesis, but not increased proliferation is further supported by other studies showing divergent roles of Msx2. Msx2 is a transcription factor that can induce cell invasion as well as proliferation via Cyclin D1 [31], [32]. However, Cyclin D1 is only a target of Msx2 when it is induced by the Wnts; Msx2 induction by the Bmps through the Smad signaling pathway does not activate Cyclin D1 [34].

Msx2 promoter is ER and PR responsive, but does not contain ERE, PRE, or GRE elements, suggesting that the effects of these hormones are indirect [7]. There have been reports that PR regulates Wnt proteins leading to Msx2 expression [8], [33], [34], while other studies point to either a cooperative effect of Wnts and Bmps [34] or to induction of Msx2 by Bmp signaling alone [31]. Our data show that Msx2 expression was not downregulated when EpH4-PR cells were exposed to the Wnt inhibitor Dkk1, but a significant decrease was observed when the cells were treated with the Bmp inhibitor Noggin [6], suggesting PR induces Msx2 expression via the Bmp pathway. Our results herein extend this initial observation and confirm that P regulates the Bmp pathway via regulation of the non-canonical BmpR type 1, Alk2.

PR cytoplasmic signaling (i.e. activation of MAPK and Src) is transient, whereas PR function as a transcription factor may take hours [33]. Therefore we investigated the regulation of PR transcriptional targets 6–24 hours post exposure to P and found that transcripts for BmpRIA/Alk3 were upregulated in EpH4-PR cells. We were unable to detect protein in the cells, although BmpRIA/Alk3 protein was readily detectable in mammary gland tissue via western analysis (*data not shown*). Others [10] have similarly reported difficulties in detecting the presence of BmpRIA/Alk3 in mouse hind limb using immunohistochemistry, suggesting a disconnect between the transcriptional and protein regulation and/or potentially rapid degradation of BmpR1A/Alk3 protein in the tissue. Indeed, the rapid internalization and degradation of BmpR1A has been reported in lymphocytes [35] and presents potential alternate regulatory mechanism not covered in the scope of the present report.

However, microarray results suggested that Alk2, a non-canonical BmpR I, was responsible for transmitting PR signaling in EpH4-PR cells. Our results confirm that Alk2 transcripts and protein are indeed upregulated in EpH4-PR cells. This occurred whether or not cells were exposed to P possibly reflecting the known unliganded action of PR-A [22]–[24]. Both canonical and non-canonical BMP signaling result in activation of Smad proteins, therefore, we focused our studies on activation of Smads. Thus, our results do not conclusively rule out the potential of other BMPR regulating Msx2. However, studies demonstrating the dominant role of Alk2 in BMP signaling suggest further studies on Alk2 signaling are warranted. For example, it was recently shown that BMP9 acts as a proliferative factor in ovarian surface epithelial cells and ovarian cancer cell lines via signaling through Alk2/Smad4 pathway, rather than through Alk1 [36]. Additionally, Endoglin, a transforming growth factor beta superfamily auxiliary receptor, was shown to inhibit prostate cancer motility via activation of the Alk2/Smad1 pathway [37], and BMP7 inhibition of epithelial-to-mesenchymal transition was lost when Alk2 were knocked down in a melanoma cell line [38]. Cross-talk between canonical and non-canonical BMP pathways may also occur by formation of a complex involving BMPR type I, Alk2, and Smad 1/5 similar to that observed in patients with fibrodysplasia ossificans progressive [39]. These cooperative effects between canonical and non-canonical BMP signaling permit transcriptional responses on specific target genes.

**Figure 6:**
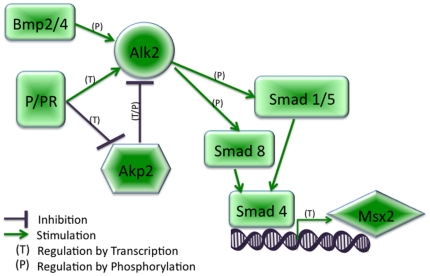
Proposed regulation of Msx2 via BMP receptor-mediated PR signaling. Progesterone receptor signaling upregulates Msx2 transcription via inhibition of the phosphatase Akp2 and upregulation of Alk2 activity, leading to phosphorylation and nuclear translocation of the Smad transcription factor proteins.

Microarray data also indicated a decrease in expression of TNAP/Akp2, an enzyme capable of hydrolyzing monophosphate esters [40] in normal and tumor tissue [19]. Among its substrates is phosphorylated Alk2 [16]. Our data indicate that when Akp2 is downregulated, Alk2 is constitutively active (caAlk2) (Fig. 6). In *Xenopus*, caAlk2 induces expression of ventral mesoderm markers reminiscent of Bmp2/4 activation [13]. In our model system, PR did not upregulate either Bmp2 or Bmp4 but upregulated Msx2 [6]. Our data show that PR downregulates Akp2 transcripts as well as Akp2 promoter activity using a luciferase reporter system, which may then result in a caAlk2. Further studies are being conducted to fully analyze the Akp2 promoter and assess whether the ½ PRE site is solely responsible for the repressive effect of P.

Akp2 downregulation and the resulting constitutive activation of Alk2 may have potentially promoted the observed upregulation of phosphorylated Smad 1,5, 8. We found that nuclear Smad 1,5, 8 were increased in EpH4-PR cells. Given that bone is a common site of breast cancer metastasis [41]–[43], it is interesting that molecules (such as BMPs, Smads, and parathyroid hormone-related protein) involved in hormonal effects on the mammary epithelial cells are relevant in bone development and perhaps tumorigenesis [44].

Our mouse mammary epithelial EpH4-PR cells constitutively overexpress Msx2 and exhibit enhanced branching [6] while overexpression of Msx2 in NMuMG mouse mammary epithelial cells resulted in epithelial-mesenchymal transition and enhanced invasiveness [32]. Additionally, immunostaining for Msx2 showed positive cells only in the infiltrating region of human infiltrating breast carcinomas; non-infiltrating tumor cells were negative for Msx2. In correlation with our observation that Alk2 regulates Msx2 expression, Alk2 was shown to stimulate endothelia-to-mesenchymal transition as a result of activating mutations in familial syndrome fibrodysplasia ossificans progressiva, and endothelial cells conditionally deficient for Alk2 fail to undergo endothelia-to-mesenchymal transition during embryogenesis [45], [46]

The relationship of P, Bmp signaling and Msx2 to breast cancer metastasis remains to be explored. Re-activation of the Akp2 gene could be helpful in downregulating aberrant Msx2 expression in PR+ breast cancers.
